# Genetic Polymorphism in PDE4D Gene and Risk of Ischemic Stroke in Chinese Population: A Meta-Analysis

**DOI:** 10.1371/journal.pone.0066374

**Published:** 2013-06-17

**Authors:** Xu Liu, Ruixia Zhu, Lei Li, Shumin Deng, Qu Li, Zhiyi He

**Affiliations:** Department of Neurology, First Affiliated Hospital of China Medical University, Shenyang, China; Cuban Neuroscience Center, Cuba

## Abstract

**Background:**

Stroke is the second most common cause of death and major cause of disability worldwide. The SNP 83 in PDE4D gene has been suggested as a risk factor in ischemic stroke, but direct evidence from genetic association studies remains inconclusive even in Chinese population.

**Methods:**

Meta-analysis of case-control studies on the relationship between SNP 83 in PDE4D gene and susceptibility to ischemic stroke in Chinese population published domestically and abroad from January 2003 to September 2012.

**Results:**

9 case-control studies were selected. Meta-analysis results showed that the significant association between SNP 83 and ischemic stroke was found under the dominant model (OR = 1.34, 95% CI: 1.20–1.49) and recessive model (OR = 1.45, 95% CI: 1.19–1.76) in Chinese population. In subgroup meta-analysis, SNP 83 and atherothrombotic stroke, rather than lacunar stroke, showed the significant association under the dominant model (OR = 1.69, 95% CI: 1.41–2.01) and recessive model (OR = 1.47, 95% CI: 1.04–2.06).

**Conclusions:**

The results suggest that SNP 83 in PDE4D gene is significantly associated with susceptibility to ischemic stroke in Chinese population.

## Introduction

Stroke is the second most common cause of death and major cause of disability worldwide. [1] In China, with 1.4 billion populations, the annual stroke mortality rate is approximately 1.6 million, which has exceeded heart disease to become the leading cause of death. In addition, China has 2.5 million new stroke cases each year and 7.5 million stroke survivors. [2] Ischemic stroke is the most type of stroke, and about 43% to 79% of all strokes are ischemic in China. [2]–[3] Traditional factors such as hypertension and smoking account for a significant proportion of ischemic stroke risk, but much risk remains unexplained. [4] Genetic risk factors, suggested by evidence from twin, case-control and cohort studies of familial aggregation, might contribute to a predisposition to ischemic stroke. [5].

The phosphodiesterase 4D (PDE4D) gene is at the locus on chromosome 5q12 and encodes cAMP-specific 30, 50-cyclic phosphodiesterase 4D, belonging to a superfamily of phosphodiesterases (PDE4 family). [6] In 2003, through genome-wide association study (GWAS), Gretarsdottir et al. [7] identify the association of SNP 83 in PDE4D gene with carotid stroke in Icelandic population. Since then, the studies on different populations have been performed to uncover whether SNP 83 in PDE4D gene could participate in ischemic stroke in non-Icelandic populations. However, based on the existing studies, the relationship between SNP 83 in PDE4D gene and ischemic stroke still remains unknown. The association between SNP 83 and ischemic stroke has been found in the population of India, Pakistan and Australia, but this association has not been replicated in the population of Italy, Germany and Japan. [8]–[13]Inconsistency among research findings might be caused by different allele frequencies across study populations, particularly in different ethnic and geographical groups. In order to reduce heterogeneity from these confounding factors, we focus our attention on the association between SNP 83 in PDE4D gene and ischemic stroke in Chinese population.

Even in Chinese population, the conclusions have not reached an agreement. Several studies have reported a positive or null relation between SNP 83 in PDE4D gene and ischemic stroke in Chinese population, but findings have been controversial. [14]–[22] The sample sizes of these studies have been relatively small. To clarify the varying results, we have undertaken this meta-analysis of SNP 83 in PDE4D gene and ischemic stroke in Chinese population.

## Materials and Methods

### Data Sources

Electronic databases including MEDLINE, CBMdisc(Chinese Biomedical Literature analysis and retrieval system for compact disc), CNKI (China National Knowledge Infrastructure), Chinese VIP database and Chinese WanFang database were searched from January 1, 2003 to September 30, 2012 for all case-control studies evaluating the association of SNP 83 in PDE4D gene with ischemic stroke in Chinese population. The following terms were used in our search strategies: PDE4D/phosphodiesterase 4D and stroke/cerebral infarction/brain infarction/cerebrovascular disease and polymorphism/gene/genotype/variant/allele. The equivalent Chinese terms were used in the Chinese databases. The “related articles” option in MEDLINE, as well as reference lists of all retrieved studies, were checked to search for other relevant articles that were not initially identified. The articles were selected without language restrictions. The entire literature search was performed by two independent researchers.

### Study Selection

The studies had to meet the following criteria: (1) The association of SNP 83 in PDE4D gene with ischemic stroke was examined based on case-control design; (2) The population of the studies was restricted to Chinese population. The studies were excluded if one of the following existed: (1) The genotype frequency was not reported, (2) For duplicate publications, the smaller dataset was discarded.

### Data Extraction

The two of the authors independently extracted data from each relevant study. Disagreements were reconciled through group discussion.

The following information was collected from each study: (1) first author, journal, year of publication, region, (2) total number, characteristics of cases and controls, subtype of ischemic stroke, distribution of genotypes and alleles in all groups.

### Statistical Analysis

Data were analyzed and processed using Review Manager 5.0 and Stata 10.0. Heterogeneity among studies was examined with the *I* ^2^ statistic and *I* ^2^>50% indicates significant heterogeneity between the studies. Based on the test of heterogeneity, a pooled OR was calculated by using fixed or random effect model, along with the 95% confidence interval (CI) to measure the strength of the genetic association. Publication bias was examined by the visual inspection of funnel plot, Begg’s test and Egger’s regression test (*p*<0.05 was considered representative of statistically significant publication bias).

## Results

### Search of Studies and Characteristics of Included Studies

**Figure 1:**
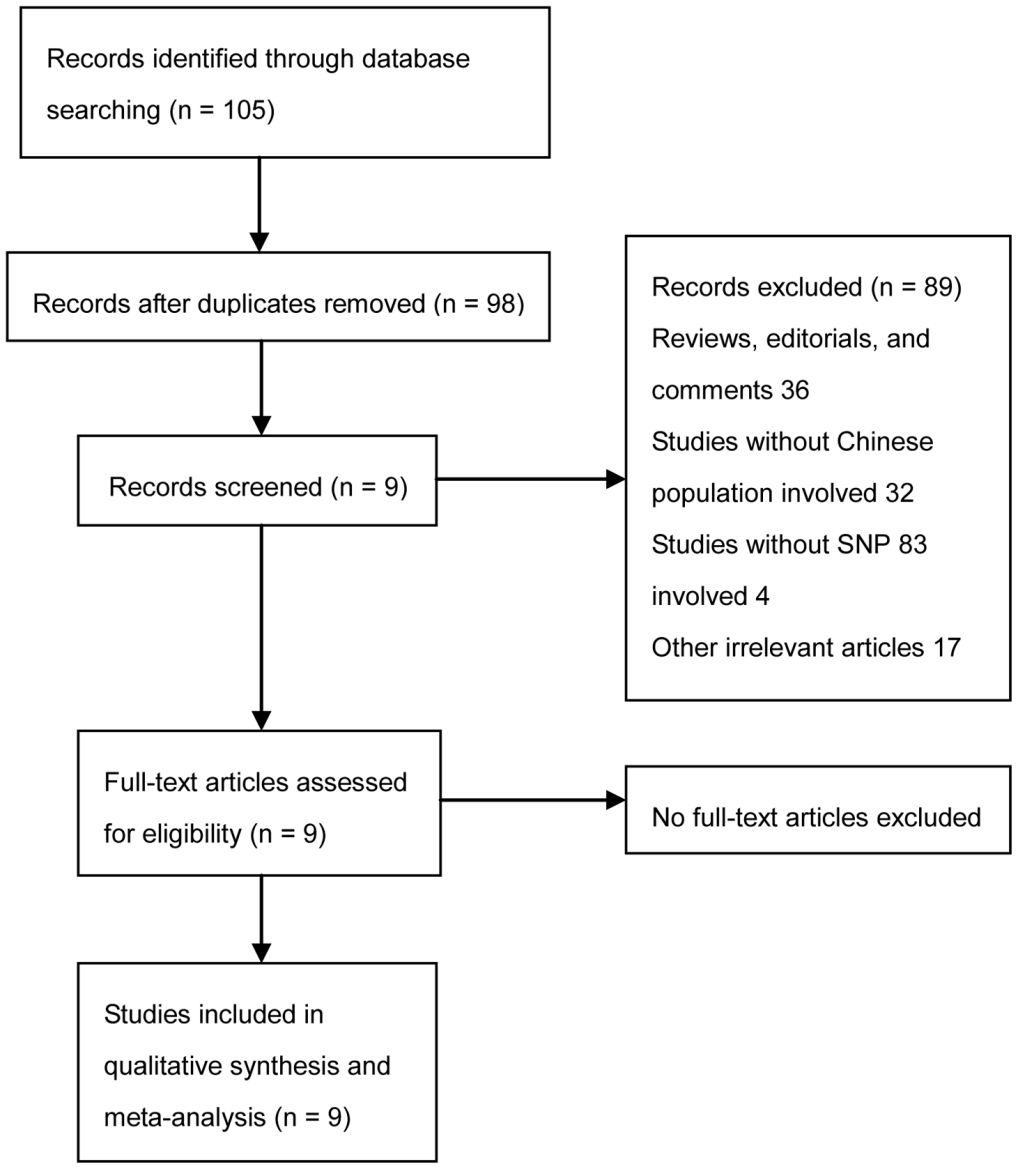
The flow diagram of study selection.

105 studies were identified by the literature search, among which 9 studies met the inclusion criteria. Of the 96 excluded studies, 7 were duplicate publication, 36 were reviews, editorials or comments, 32 were from other ethnic populations, 4 were not involved with SNP 83, 17 were other irrelevant articles. A flow diagram schematized the process of selecting and excluding articles with specific reasons, as shown in Figure 1.

A total of 9 case-control studies of Chinese population were included in the meta-analysis comprising 2751 patients with ischemic stroke and 3233 controls. The ischemic stroke in most studies of our meta-analysis was confirmed by CT or MRI, except one study in which the definition of stroke was not mentioned. [22]The detailed characteristics of the included studies were shown in Table 1. The distributions of genotypes in the individual studies for SNP 83 were shown in Table 2.

**Table 1:** Characteristics of included studies.

1^st^ author, year	Region	Characteristics of the included studies	
		Ischemic stroke group	Control group
Li C, 2012	Shandong	N = 440, Age: 66.6±8.4 years, Male: 66.1%, ischemic stroke (LAA, SVO, CE)	N = 486, Age: 66.1±5.2 years, Male: 64.6%
Wang HM, 2012	Zhejiang	N = 235, Age: 61.3±10.1 years, Male: 60.9%, atherothrombotic ischemic stroke	N = 105, Age: 60.0±9.4 years, Male: 57.1%
Cheng H, 2011	Jiangsu	N = 280, Age: 59.6±12.4 years, Male: 62.9%, lacunar stroke	N = 258, Age: 58.4±13.6 years, Male: 60.5%
Li N, 2010	Liaoning	N = 371, Age: 63.9±7.4 years, Male: 62.0%, ischemic stroke	N = 371, Age: 62.9±7.6 years, Male: 66.6%
Sun Y, 2009	Shanghai	N = 649, Age: 73.2±9.4 years, Male: 56%, ischemic stroke (LAA+CE, SVO)	N = 761, Age: 73.3±7.3 years, Male: 55%
Xue H, 2009	Shanxi, Chongqing,Beijing, Tianjin	N = 424, No description of age and gender, ischemic stroke (LAA, SVO)	N = 887, Age: 60.7±8.2 years, Male: 57.6%
Zhang HL, 2009	Heilongjiang	N = 60, No description of age and gender, ischemic stroke (LAA, SVO)	N = 44, Age: 58.6±17.6 years, Male: 47.7%
Xu SL, 2008	Jiangsu	N = 116, Age: 65.9±12.4 years, Male: 60.3%, ischemic stroke (LAA, SVO)	N = 110, Age: 65.1±12.7 years, Male: 54.5%
Lin HF, 2007	Taiwan	N = 190, No description of age and gender, early onset ischemic stroke	N = 211, No description of age and gender

Abbreviations: LAA: large-artery atherosclerosis; SVO: small-vessel occlusion; CE: cardioembolism.

**Table 2:** Distribution of genotypes in the individual studies**.**

1^st^ author, year	Case			Control			HWE for Controls	
	CC	CT	TT	CC	CT	TT	?^2^	*P*
Li C, 2012	48	182	210	39	170	277	3.09	0.079
Wang HM, 2012	16	82	137	4	25	76	1.08	0.300
Cheng H, 2011	12	102	166	12	94	152	0.28	0.598
Li N, 2010	117	173	81	76	197	98	1.60	0.205
Sun Y, 2009	40	223	385	35	230	496	1.55	0.213
Xue H, 2009	27	144	253	29	255	603	0.10	0.749
Zhang HL, 2009	3	20	37	4	10	30	4.00	0.045
Xu SL, 2008	4	46	66	6	29	75	1.88	0.171
Lin HF, 2007	6	54	117	13	51	147	7.60	0.006

Abbreviations: HWE: Hardy-Weinberg equilibrium.

### Results of Meta-analysis

**Figure 2:**
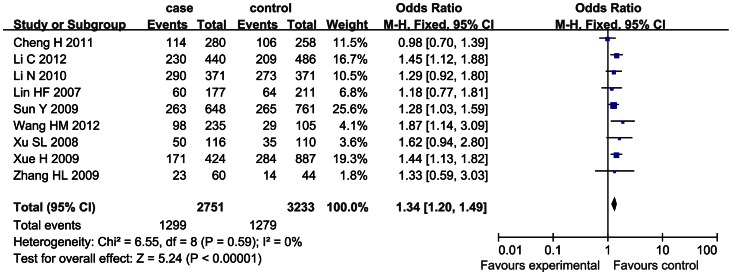
Forrest plot on the association between SNP 83 and ischemic stroke under the dominant model (CC+CT/TT).

**Figure 3:**
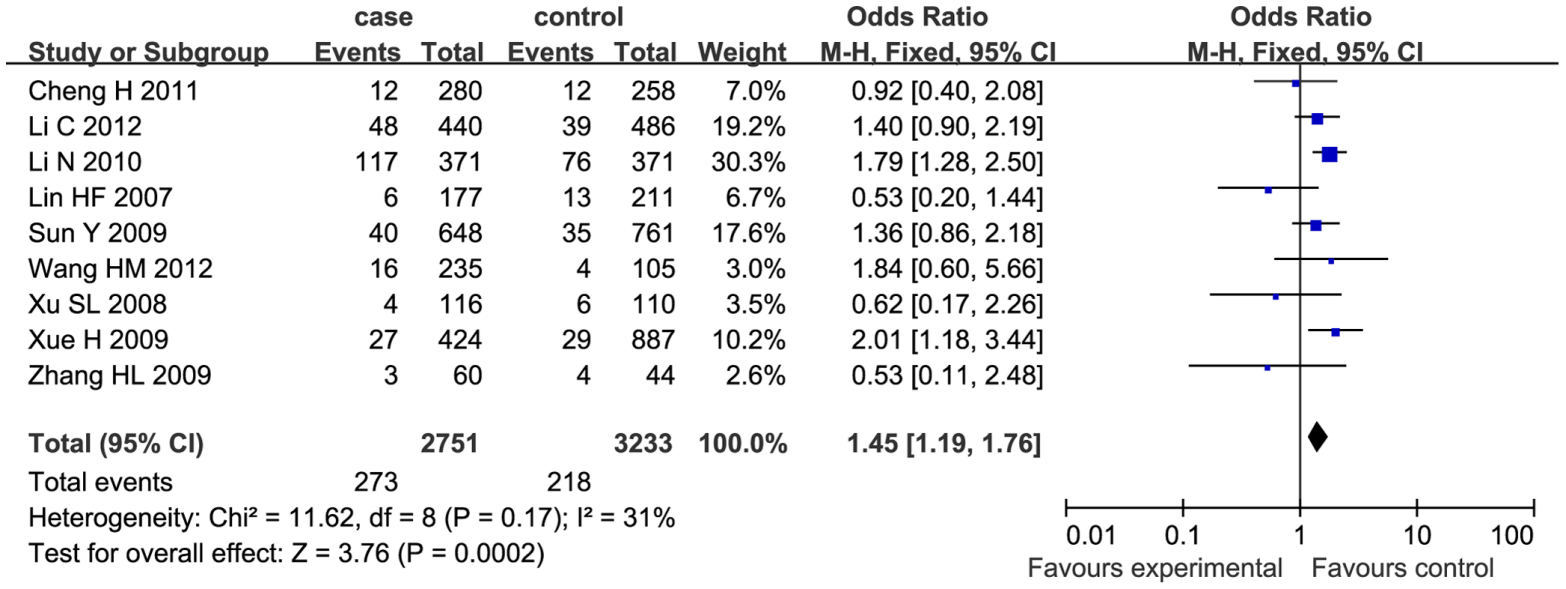
Forrest plot on the association between SNP 83 and ischemic stroke under the recessive model (CC/CT+TT).

For SNP 83, the heterogeneity among the studies, measured by *I^2^* statistic, was not significant under the dominant model (*I^2^* = 0) and recessive model (*I^2^* = 31%). Therefore, the fixed effect model was used for calculating the pooled OR for the both models. A significant association between SNP 83 and ischemic stroke was found under the dominant model (OR = 1.34, 95% CI: 1.20–1.49, *p*<0.00001) and recessive model (OR = 1.45, 95% CI: 1.19–1.76, *p* = 0.0002), as shown in Figure 2 and 3.

### Sensitivity Analysis

**Figure 4:**
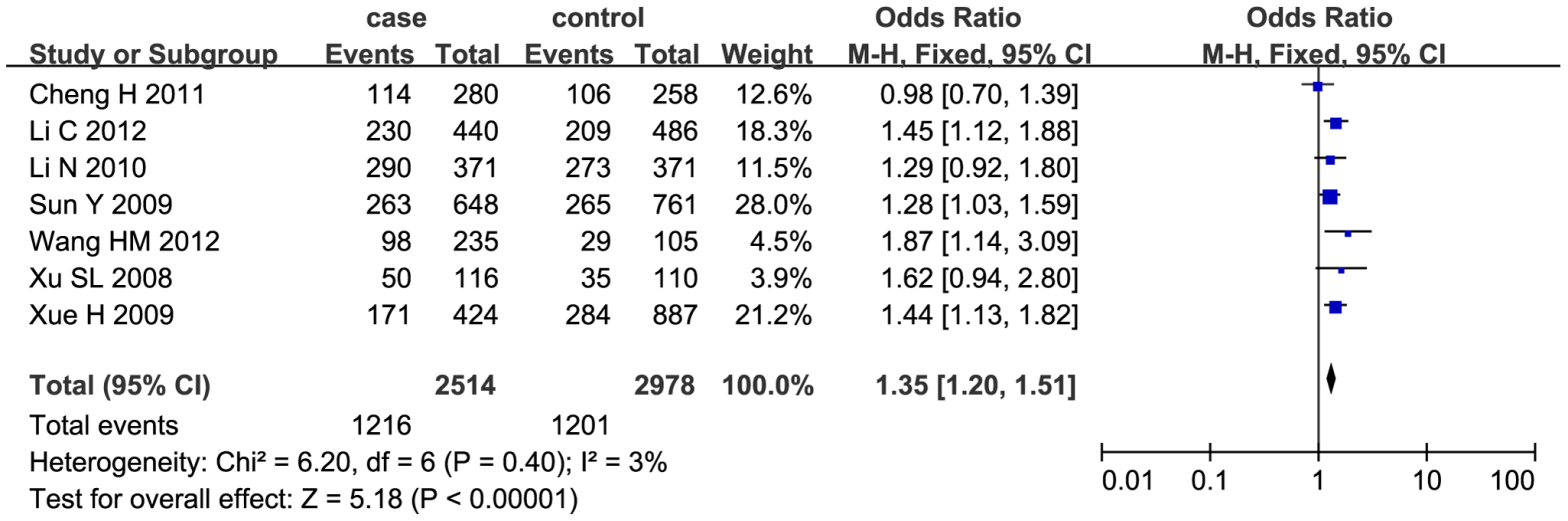
Sensitivity analysis on the association between SNP 83 and ischemic stroke under the dominant model(CC+CT/TT).

**Figure 5:**
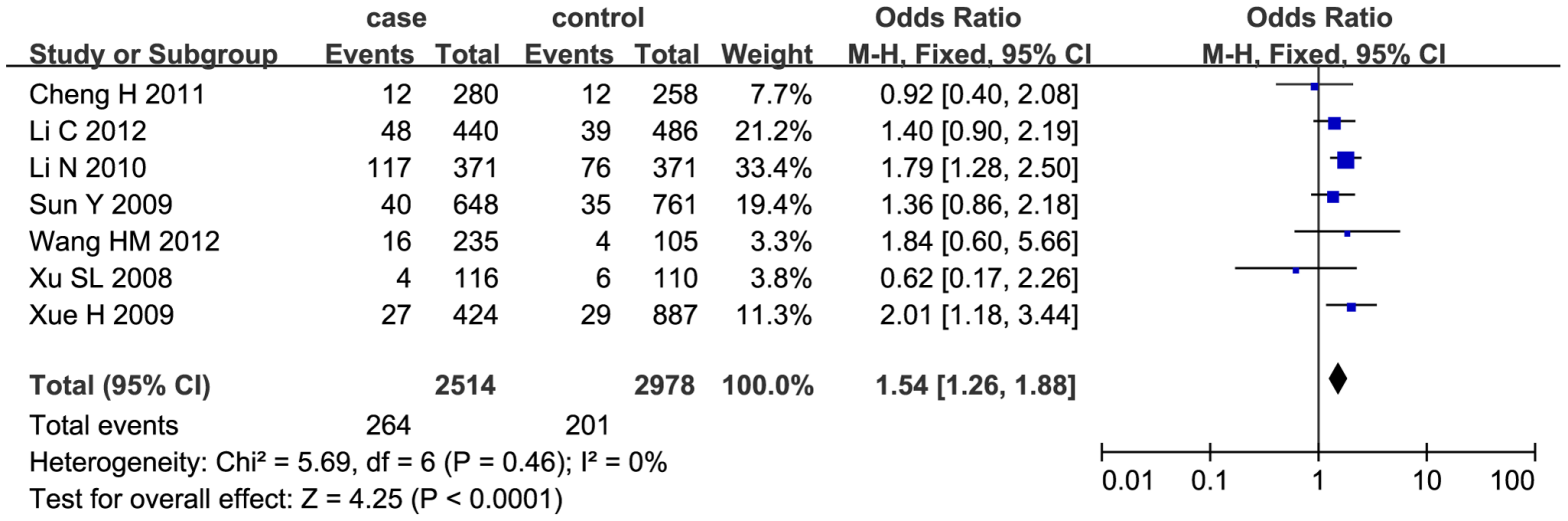
Sensitivity analysis on the association between SNP 83 and ischemic stroke under the recessive model (CC/CT+TT).

The sensitivity analysis was performed with 7 case-control studies whose genotype frequencies in the control group were consistent with Hardy-Weinberg equilibrium (HWE). [14]–[19], [21] The heterogeneity between studies was not significant (*I* ^2^<0.5) and the fixed effect model was used for calculating the pooled OR. The sensitivity analysis demonstrated that the significant positive association between SNP 83 and ischemic stroke was found under the dominant model (OR = 1.35, 95% CI: 1.20–1.51, *p*<0.00001) and recessive model (OR = 1.54, 95% CI: 1.26–1.88, *p*<0.0001), as shown in Figure 4 and 5.

### Subgroup Meta-analysis

**Figure 6:**
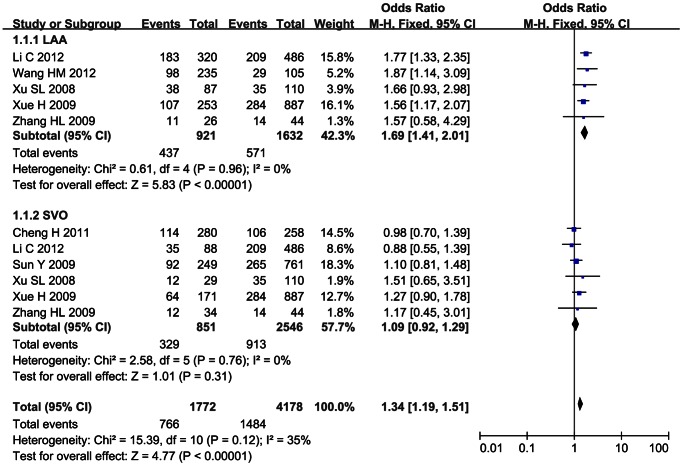
Subgroup analysis on the association between SNP 83 and ischemic stroke under the dominant model (CC+CT/TT).

**Figure 7:**
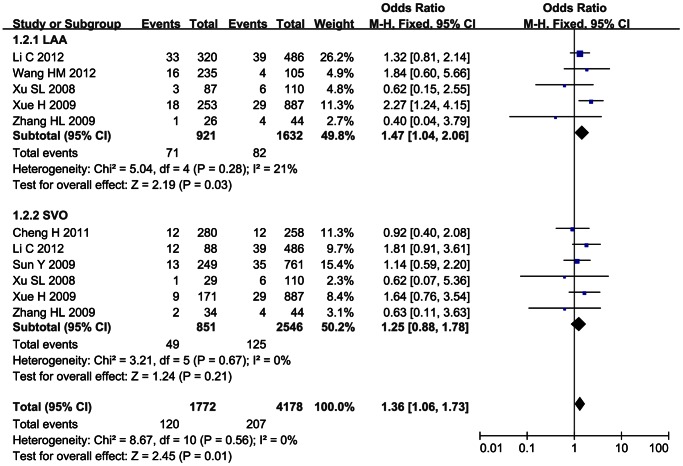
Subgroup analysis on the association between SNP 83 and ischemic stroke under the recessive model (CC/CT+TT).

Subgroup analysis was performed for SNP 83 in PDE4D gene and ischemic stroke in Chinese population by the subtypes of ischemic stroke. 5 studies were selected in the subgroup of large-artery atherosclerosis (LAA) [14]–[15], [19]–[21], 6 studies in the subgroup of small-vessel occlusion (SVO) [14], [16], [18]–[21]. The association between SNP 83 and ischemic stroke was significant in LAA subgroup under the dominant model (OR = 1.69, 95% CI: 1.41–2.01, *p*<0.00001) and recessive model (OR = 1.47, 95% CI: 1.04–2.06, *p* = 0.03). The remaining pooled OR from SVO subgroup was not significant under the dominant or recessive model (*p*>0.05), as shown in Figure 6 and 7.

### Publication Bias

**Figure 8:**
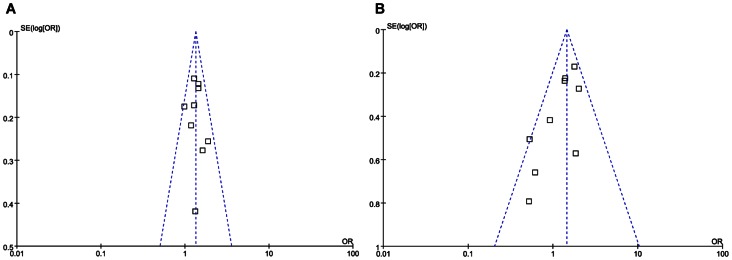
Funnel plot for studies investigating the effect of SNP 83 on the risk of ischemic stroke under the dominant (pane A) and recessive (pane B) models.

For SNP 83,the distribution of the ORs from individual studies in relation to their respective standard deviation in funnel plot, as shown in Figure 8, failed to detect significant bias under the dominant model, but not recessive model. By using Begg’s test, there was no statistical evidence of publication bias among studies (*p* = 0.602 for dominant model and *p* = 0.076 for recessive model, respectively). In Egger’s regression test, there was no statistical evidence of publication bias under the dominant model, but not for the recessive model (*p* = 0.754 for dominant model and *p* = 0.026 for recessive model, respectively).

## Discussion

Case-control studies are commonly used to find the association between the candidate genes and the complex human diseases. However, the data from the genetic association studies indicates that susceptibility loci for common disease have a small effect. This means that genetic association studies need to test many cases and controls to have a reasonable chance of finding an effect. [23] By increasing the sample size, the meta-analysis has the potential to detect small effects in human genetic association studies. Therefore, we have conducted this meta-analysis in order to derive a more convinced result between SNP 83 in PDE4D gene and susceptibility to ischemic stroke in Chinese population.

Our meta-analysis showed that the risk of developing ischemic stroke in carriers with C allele on SNP 83 was 1.34-fold higher than individuals without C allele in Chinese population. Also, the risk in individuals with CC genotype was 1.45-fold higher than individuals without CC genotype. In case-control studies, a control group that is representative of the general population is expected to be in HWE for the studied locus. Therefore, we selected the case-control studies whose genotype frequencies in the control group were consistent with HWE for sensitivity analysis. The results of sensitivity analysis were close to the results of the meta-analysis before the sensitivity analysis. So it suggests that the combined results of our meta-analysis are reliable.

In 2008, Bevan et al. [24] performed a similar meta-analysis of 9 studies. They did not find any association between SNP 83 and ischemic stroke under the dominant model (OR = 0.96, 95% CI: 0.87–1.07) or recessive model (OR = 0.97, 95% CI: 0.85–1.10). In Bevan’s meta-analysis, the majority of subjects in studies were white, while the population of the studies in our meta-analysis was restricted to Chinese population. The population difference of two meta-analyses may help to explain the difference of Bevan’s results and ours. In 2010, Xu et al. [25] conducted another similar meta-analysis of 7 studies on Asian populations, subjects in 3 of which were Chinese population. The association between PDE4D and ischemic stroke was confirmed for allele C homozygote of SNP 83 (OR = 1.42, 95% CI: 1.14–1.77) but not in the carriers (OR = 1.20, 95% CI: 0.97–1.47). The risk of ischemic stroke in individuals with CC genotype in our meta-analysis was similar to the risk in Xu’s study of Asian populations. Notably, as for carriers with C allele on SNP 83, we also found a significant association with ischemic stroke which was different from Xu’s result.

How can we explain the association between SNP 83 in PDE4D gene and ischemic stroke in Chinese population? 1) PDE4D selectively degrades the second messenger cyclic adenosine monophosphate (cAMP) in vascular smooth muscle cells and activated macrophages, which is a key signaling molecule mediating cell proliferation and migration related to atherosclerosis and plaque stability. [26]–[29] 2) cAMP has been reported to be responsible for neuron survival and a selective inhibitor of cAMP-specific phosphodiesterase type 4 (PDE4) can promote the survival of newborn hippocampal neurons after ischemia.[30]–[32] 3) SNP 83, which is located at the 5′ end of PDE4D, may affect the transcription, splicing, message stability, or message transport of one or more isoforms. [7].

According to TOAST classification, ischemic stroke can be divided into five subtypes: 1) LAA, 2) SVO, 3) cardioembolism (CE), 4) stroke of other determined etiology, and 5) stroke of undetermined etiology. [33] In Chinese population, five studies explored the association between SNP 83 and LAA, and six explored the association between SNP 83 and SVO. Hence, the meta-analysis was conducted in these two subgroups. However, we found the significant association of SNP 83 with ischemic stroke only in LAA subgroup. That can be explained by the role of PDE4D gene in the process of atherosclerosis, as mentioned above. As for the other three subtypes of ischemic stroke, insufficient data were available for conducting subgroup meta-analysis.

The present meta-analysis should be interpreted within the context of its limitations. 1) All included studies of our meta-analysis have followed a retrospective case-control design. It may be more subject to bias and artifact than prospective studies. 2) If another variant in or near the SNP 83 in PDE4D gene was the causal variant, the true association could easily be missed. Different linkage disequilibrium patterns with the causal variant may lead to variable results in different populations. 3) We only focused on SNP 83 in PDE4D gene, while did not evaluate other genes or environmental factors. It is possible that the potential roles of SNP 83 in PDE4D gene are diluted or masked by other gene-gene or gene-environment interactions. 4) Publication bias refers to the decreased likelihood of studies’ results being published when they are near the null, not statistically significant. Publication bias is one of the most important sources of bias in meta-analysis, which can cause the false positive results. In our meta-analysis, under the dominant model, we found no evidence of publication bias by the visual inspection of funnel plot, Begg’s test or Egger’s regression test. However, under the recessive model, we found publication bias by the visual inspection of funnel plot and Egger’s regression test. Hence, the caution is indicated when interpreting the association of SNP 83 with ischemic stroke under the recessive model.

In conclusion, our meta-analysis suggests that SNP 83 in PDE4D gene is associated with developing ischemic stroke in Chinese population. Given the limitations in the present meta-analysis, the results need to be interpreted with caution. Well-designed case-control studies with large sample sizes regarding the association of SNP 83 in PDE4D gene and ischemic stroke need to be performed on different ethnic population in the future.

## Supporting Information

File S1 **PRISMA 2009 Checklist.**
(DOC)
